# Case report: Persistent ST-segment elevation due to cardiac metastasis from lung cancer

**DOI:** 10.3389/fcvm.2023.1001527

**Published:** 2023-02-08

**Authors:** Jiawei Zhou, Chengchuang Zhan, Jing Zhou, Chao Wei, Cao Zou

**Affiliations:** ^1^Department of Cardiology, The First Affiliated Hospital of Soochow University, Suzhou, China; ^2^Department of Echocardiography, The First Affiliated Hospital of Soochow University, Suzhou, China

**Keywords:** cardio-oncology, cardiac metastasis, lung cancer, ST-segment elevation, case report

## Abstract

Patients with secondary cardiac cancer occasionally show ST segment elevation that mimics acute coronary syndrome despite the absence of coronary artery occlusion. We herein describe a rare case of secondary cardiac cancer that presented with ST-segment elevation. An 82-year-old Chinese man was admitted to the hospital with chest discomfort. Electrocardiography (ECG) showed ST segment elevation in the precordial leads and low-voltage QRS complexes in limb leads without the development of *Q* waves. Unexpectedly, emergency coronary angiography showed no significant stenosis of the coronary arteries. However, fortunately, transthoracic echocardiography (TTE) revealed massive pericardial effusion and a mass at the apex of the ventricular myocardium. Coincidentally, contrast-enhanced chest computed tomography showed primary lung cancer in the left lower lobe, pericardial effusion, and myocardial metastasis at the ventricular apex. The pericardiac fluid contained blood with significantly increased CEA levels and exfoliated tumor cells. The lung histopathological report suggested squamous cell carcinoma. Two months later, the patient died. These findings suggested that the persistent ST-segment without the development of Q waves was associated with ventricular invasion by primary lung cancer and may indicate a poor prognosis. In conclusion, physicians should be aware of persistent ST-segment elevation mimicking myocardial infarction due to cardiac metastasis with a poor prognosis.

## Introduction

Cardiac tumors comprise primary tumors, including benign and malignant, metastatic tumors, and some other tumor-like camouflages (such as thrombosis) (1). Heart-related clinical manifestations often include dyspnea, arrhythmia, obstruction, heart failure, and systemic embolism. Related auxiliary examinations mainly include electrocardiogram (ECG), echocardiography, computed tomography (CT), cardiac magnetic resonance (CMR), and related laboratory tests. As a convenient, rapid, and intuitive examination, echocardiography often plays the primary role in finding lesions, confirming the extent of the lesions, and assessing cardiac function. Contrast-enhanced CT and cardiac magnetic resonance can further analyze the nature of the lesion, identify the tumor source, and display the extent of the invasion. Herein, we describe a case of cardiac metastasis from lung cancer accompanied by massive pericardial effusion, which was comprehensively evaluated by ECG, echocardiography, contrast-enhanced CT, laboratory tests, and histopathological assessment.

## Case description

On 9 October 2021, an 82-year-old man presented at the emergency department with an 8-h history of chest discomfort, nausea, vomiting, and orthopnea. He reported worsening chest discomfort during exertion accompanied by nausea, anorexia, and fatigue without any cough, chest pain, or edema of the lower limb for 20 days. His medical history included hypertension, hyperlipidemia, diabetes mellitus, and former tobacco use for 50 years.

**FIGURE 1:**
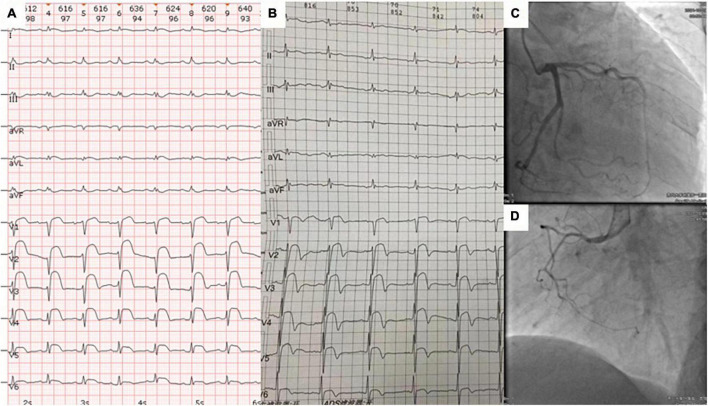
The ECG at admission **(A)** and 4 months prior to admission **(B)** showed abnormal ST segment elevations in leads V1–V6 and low-voltage QRS complexes in limb leads. Coronary angiography revealed no significant stenosis in the right coronary, left anterior descending, and circumflex coronary arteries **(C,D)**.

On presentation, the patient’s blood pressure was 123/72 mmHg, heart rate was 90 beats/min, oxygen saturation was 98%, and respiratory rate was 18 breaths/min. A cardiovascular physical examination showed a decreased S1 with no murmurs, rubs, or gallops. Respiratory and abdominal examinations were also within normal limits. ECG showed sinus tachycardia, ST segment elevation in precordial leads V1–V6, and low-voltage QRS complexes in limb leads (Figure 1A). Laboratory studies were notable for high sensitivity troponin T (hs-TnT) of 21.08 pg/ml (normal < 14 pg/ml) and *N*-terminal (NT)-pro hormone BNP (NT-proBNP) of 2,160 pg/ml (normal < 125 pg/ml). Acute precordial STEMI diagnosis was considered. We performed emergent coronary angiography, but there was no significant stenosis of coronary arteries (Figures 1C, D). Therefore, myocardial infarction of the non-obstructive coronary artery (MINOCA) was considered.

**FIGURE 2:**
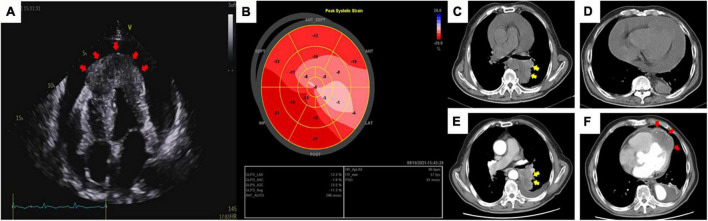
Transthoracic echocardiography **(A)** revealed massive pericardial effusion and a thickened and hypoechoic mass (red arrow) at the apex of the heart *via* an apical four-chamber view. Global longitudinal strain **(B)** showed a significantly decreased absolute value of strain in the area of the anterior and lateral walls of the heart. Routine chest CT at admission **(C,D)** and contrast-enhanced chest CT after pericardiocentesis **(E,F)** showed an irregular iso-density mass (yellow arrow) with peripheral exudation in the left lower lobe of the lung panels **(C,E)** and a myocardial mass (red arrow) located in the left and right ventricular apex emerging with reduced pericardial effusion panel **(F)**.

On day 2, a transthoracic echocardiogram (TTE) revealed massive pericardial effusion and a 6.5 × 3.1 cm-sized hypoechoic mass at the apex of left ventricular, septal, and right ventricular myocardium associated with hypokinesis and decreased absolute value of global longitudinal strain (Figures 2A, B). Contrast-enhanced chest CT showed scattered irregular iso-density shadow (the largest mass was 67 × 47 mm) with peripheral exudation in the left lower lobe and significant thickness at the ventricular apex with pericardial effusion (Figures 2C–F). Tumor marker levels were elevated as follows: CEA of 32.4 ng/ml (normal < 10 ng/ml), CA125 of 99.8 U/ml (normal < 35 U/ml), TPSA of 29.9 ng/ml (normal < 4 ng/ml), CYFRA211 of 57.4 ng/ml (normal < 3.07 ng/ml), and SCCA of 39.6 ng/ml (normal < 1.5 ng/ml).

**FIGURE 3:**
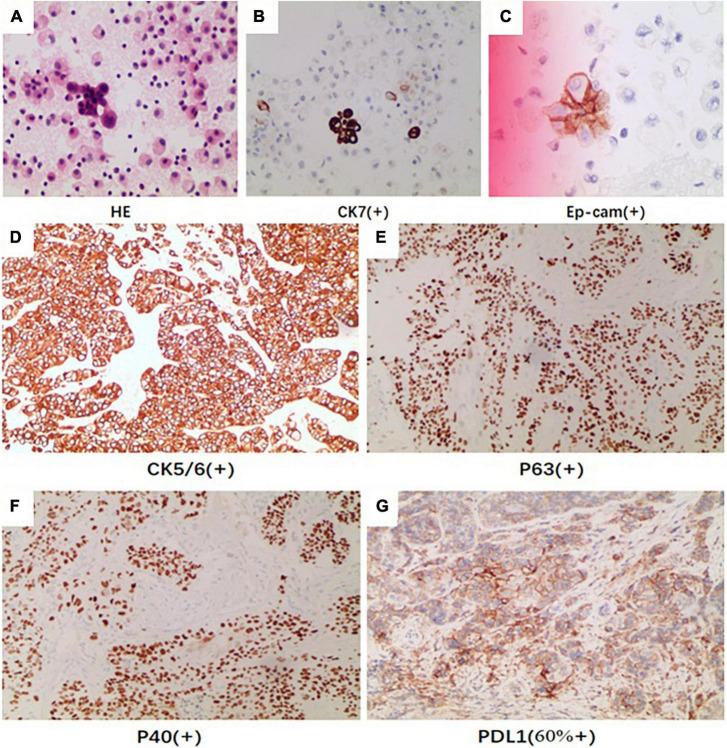
The pericardiac fluid by HE revealed **(A)** exfoliated tumor cells with **(B)** CK7 (positive) and **(C)** Ep-cam (positive). The immunohistochemical analysis of pathologic tissue by percutaneous lung puncture showed **(D)** CK5/6 (positive), **(E)** P40 (positive), **(F)** P63 (positive), and **(G)** PDL1 (positive).

**FIGURE 4:**
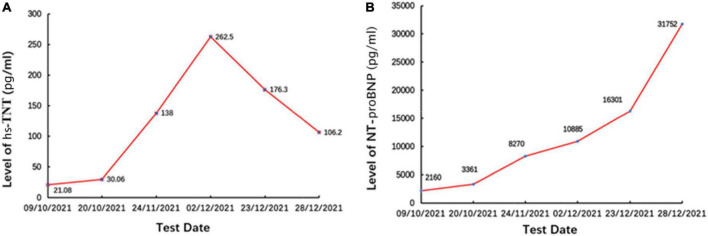
The levels of **(A)** hs-TNT and **(B)** NT-proBNP. Panel **(B)** were persistently increased, demonstrating decreased cardiac function.

On day 3, we performed pericardiocentesis. The pericardiac fluid contained blood with significantly increased CEA level (>1,000 ng/ml, normal < 5 ng/ml) and exfoliated tumor cells with CK7(+) and Ep-cam(+) (Figures 3A–C). When we obtained his medical history, his family provided an ECG performed 4 months before (Figure 1B), which demonstrated ST elevation and *T*-wave inversion similar to the ECG on this admission. These findings strongly suggested ventricular myocardial metastasis of primary lung cancer. We recommended that the patient should undergo a transbronchial lung biopsy to obtain a histopathological diagnosis, and he accepted it. One week later, he was transferred to the respiratory department. During hospitalization in the respiratory department, the patient underwent a percutaneous lung puncture. The lung histopathological report suggested squamous cell carcinoma. Immunohistochemical analyses showed CK5/6(+), P63(+), P40(+), and PD-L1 (22C3, about 60% +) (Figures 3D–G). Squamous cell carcinoma of the lungs at the stage of IVB (T3N1M1c) was considered a diagnosis. He was treated with immunotherapy by Sintilimab (PD-1 monoclonal antibody). Unfortunately, his cardiac function decreased with increased levels of hs-TNT and NT-proBNP (Figures 4A, B), and without any improvement. At the end of December 2021, he died of respiratory and heart failure. An autopsy could not be performed.

## Discussion

Persistent ST-segment elevation (STE) newly found in patients should usually be considered STEMI. However, in our case, there was abnormal ST-segment elevation on precordial leads without the development of *Q* waves different from the typical ECG characteristics of STEMI. Several cases of cardiac metastasis mimicking STEMI have been reported (2–5). ECG findings of localized STE without *Q* waves have high specificity for myocardial tumor invasion (6).

In most cases of previously reported cardiac metastases with STE, cardiac enzymes have been normal (5), unlike in our patient. The present case was initially misdiagnosed as STEMI due to STE accompanied by an elevation in hs-TNT. However, emergent CAG did not reveal any significant coronary stenosis to explain the ECG and hs-TNT abnormalities. During hospitalization, the assessment of this patient also showed increased levels of hs-TNT and NT-proBNP due to aggravated myocardial injury for a long time because a previous ECG had demonstrated similar abnormal STE 4 months before admission.

The mechanism of STE in cardiac metastasis has not been fully understood. Some case reports describe STE caused by myocardial metastases (7–10), coronary artery invasion or direct compression, or pericardial involvement (11). The most frequent mode of metastasis is *via* the lymphatic pathway, followed by hematogenous spread (12). In our case, echocardiography was the initial imaging modality to detect pericardial effusion and the presence of cardiac metastasis. Then, contrast-enhanced chest CT revealed lung cancer in the left lower lobe and cardiac abnormality at the ventricular apex without any evidence of coronary artery invasion or direct compression. In addition, there were exfoliated tumor cells and significantly increased CEA levels in the patient’s pericardiac fluid. Therefore, STE in this patient was due to myocardial injury caused by lung cancer metastases.

Lung cancer is the most frequent cause of cardiac metastatic tumors, with a reported incidence of up to 25% at autopsy, and squamous cell carcinoma is the most frequent cause of secondary cardiac cancer (13). In general, the pericardium is the most common cardiac site for metastasis *via* retrograde lymphatic extension from the lung to the heart (14, 15). In our patient, the lung histopathological report suggested squamous cell carcinoma, and echocardiography and chest CT detected massive pericardial effusion and myocardial metastatic lesions in the apical areas, respectively, which receive their blood supply from the left anterior descending artery. Therefore, our patient had STE in the precordial leads, which was similar to a case in which STE reflects the location of STEMI due to an obstruction of the left anterior descending artery.

Cardiac metastases have a poor prognosis. A myocardial metastatic lesion is often clinically silent, although it can cause malignant pericardial effusion with or without symptoms of pericarditis, arrhythmias, heart failure, and rarely acute myocardial infarction (14, 16, 17). This feature results in misdiagnosis or diagnosis delay in severe conditions. When the first ECG of our patient was recorded, STE was not treated. Four months later, he had symptoms and was admitted to our hospital. During hospitalization, his condition worsened, his cardiac function decreased, and he died of respiratory and heart failure, although he received immunotherapy. Radical surgical resection, radiotherapy, and chemotherapy could be useful treatments for certain cardiac metastases. Unfortunately, our patient had no further choice.

The changes in ECG, such as STE that mimics acute coronary syndrome, caused by secondary cardiac cancer mentioned in this article, are not the first reported, and many related studies (2–8) have recognized this phenomenon before. However, this is not just another example of STEMI misdiagnosis. Our purpose of the case report was as follows. First, we aimed to stress the significance that our physicians should consider the possibility of cardiac metastasis in patients who are first diagnosed with acute coronary syndrome (ACS). Second, we wanted to show a complete diagnostic process of this case and list the relevant examination results.

It can be asked why coronary angiography was performed without echocardiography. Actually, it is a common phenomenon in China. The main reason is that emergency procedures are different. Once patients develop symptoms of significant chest tightness, pain accompanied by STE in precordial leads, including V1–V6, on ECG, and a significant increase in troponin, they will be diagnosed with ACS and undergo emergency percutaneous coronary intervention (PCI) as quickly as possible. Echocardiography is often unnecessary in the first place. However, no severe stenosis was found during the operation; hence, MINOCA was considered. This is common sense in emergency procedures in China. Not all patients who are primarily diagnosed with ACS undergo echocardiography before emergency coronary angiography.

After we repeatedly confirmed the patient’s physical signs, we did not find distention of the jugular veins but confirmed the distention of the inferior vena cava by echocardiography. This finding might be related to the chronic course of the disease rather than acute cardiac tamponade. Conversely, engorged jugular veins are normally more pronounced in older and leaner people without any discomfort.

## Conclusion

When physicians treat patients with STE, they should first consider the diagnosis of ACS. However, when clinicians further evaluate the history and find that STE persists for a few days or more, especially with a history of tumor, they should consider the special situation described in this case report. Although the incidence of persistent STE due to cardiac metastasis is low, considering its possibility can help us broaden the horizon of the diagnosis process and head in the right direction quicker. We should also be aware of the poor prognosis of patients and try to inform patients and their families of the severity of the disease in advance. The significance of this case report lies in broadening our diagnostic thinking and providing a relatively complete and feasible diagnostic process for cardiac metastatic tumors.

## Data availability statement

The original contributions presented in this study are included in the article/Supplementary material, further inquiries can be directed to the corresponding author.

## Ethics statement

Written informed consent was obtained from the individual(s) for the publication of any potentially identifiable images or data included in this article. Written informed consent was obtained for the publication of this case report.

## Author contributions

JiaZ and CZh were responsible for collecting case data, analyzing data, and writing manuscript. CZo was responsible for review, submission, and modification of manuscript. JinZ and CW were responsible for patient’s diagnosis and treatment. All authors contributed to the article and approved the submitted version.
